# Exosomal biomarkers in the differential diagnosis of ovarian tumors: the emerging roles of CA125, HE4, and C5a

**DOI:** 10.1186/s13048-023-01336-6

**Published:** 2024-01-05

**Authors:** Huihui Shi, Liya Liu, Xueli Deng, Xiaoyu Xing, Yan Zhang, Yemeli Djouda Rebecca, Liping Han

**Affiliations:** 1 Department of Gynecology, The First Affiliated Hospital of Zhengzhou University, Henan, Zhengzhou, 450000 China; 2 1 East Jianshe Road, Zhengzhou, 450052 China

## Abstract

**Objective:**

Investigating the utility of serum exosomal markers CA125, HE4, and C5a, both individually and in combination, for distinguishing between benign and malignant ovarian tumors.

**Methods:**

In this study, we selected a total of 234 patients diagnosed with ovarian tumors, including 34 with malignant tumors, 10 with borderline ovarian tumors, and 190 with benign tumors. This study conducted comparisons of exosomal levels of CA125, HE4, and C5a among distinct groups, as well as making comparisons between serum and exosomal levels of CA125 and HE4. Furthermore, the diagnostic performance was assessed through Receiver Operating Characteristic (ROC) curve analysis. The Area Under the Curve (AUC) was computed, and a comparative evaluation of sensitivity and specificity was conducted to ascertain their effectiveness in determining the nature of ovarian tumors across different markers.

**Results:**

Serum CA125 and HE4 levels, the ROMA index, exosomal CA125, HE4, C5a levels, and their combined applied value (OCS value) were notably elevated in the ovarian non-benign tumor group compared to the benign tumor group, with statistical significance (*P* < 0.05). Exosomal and serum levels of CA125 and HE4 exhibited a positive correlation, with concentrations of these markers in serum surpassing those in exosomes. The combined OCS (AUC = 0.871) for CA125, HE4, and C5a in exosomes demonstrated superior sensitivity (0.773) and specificity (0.932) compared to serum tumor markers (CA125, HE4) and the ROMA index. The tumor stage represents an autonomous risk factor influencing the prognosis of individuals with ovarian malignancies.

**Conclusion:**

The stage of ovarian malignancy is an independent risk factor for its prognosis. The combination of exosomal CA125, HE4 and C5a has a higher clinical value for the identification of the nature of ovarian tumours.

## Introduction

According to the statistical data released in the United States in 2023,ovarian cancer is positioned as the third most prevalent among newly diagnosed tumors in the female reproductive system. Additionally, it holds the highest rank in terms of estimated mortality rates. Moreover, ovarian cancer stands as the fifth leading cause of cancer-related deaths in women [1]. Ovarian cancer typically manifests with few to no symptoms during its early stages, and the disease advances rapidly, resulting in diagnosis at an advanced stage in over 70% of patients [2]. The overall prognosis for individuals with ovarian cancer is unfavorable, as indicated by a five-year survival rate of only 46% [3]. Furthermore, the prognosis of ovarian cancer patients is intricately linked to their initial stage of diagnosis. For patients with stage I or II ovarian cancer, the five-year survival rate exceeds 70%, while for those with stage III or IV ovarian cancer, it ranges between 20 and 40% [4, 5]. Due to a growing emphasis on physical examinations, an increasing number of ovarian tumor patients are being identified during asymptomatic stages. Likewise, there has been a rise in the incidence of unnecessary surgeries for ovarian tumors, which, in turn, increase the risk of premature ovarian failure and infertility caused by surgical procedures [6, 7]. Hence, it becomes especially crucial to possess high-sensitivity indices for distinguishing between benign and malignant ovarian tumors before proceeding with surgery. Currently, the characterization of ovarian tumors depends on the integration of serum markers and radiological imaging.

Exosomes, a type of extracellular vesicle, have a diameter typically ranging from 30 to 100 nm and are released by various cells in the human body. They contain a variety of biologically active molecules, including proteins, lipids, DNA, and RNA. Exosomes play a crucial role in important processes such as tumorigenesis and disease diagnosis by modulating gene expression and protein translation [8].Research has increasingly highlighted the pivotal role of exosomes in facilitating tumor growth, including activities such as angiogenesis, immune response modulation, and the promotion of tumor metastasis [9]. The disparities in the quantity of exosomes and the composition and content of their cargo between healthy individuals and ovarian cancer patients indicate that they may serve as a novel biomarker for the early detection of the disease [10]. Exosomal proteins originating from cancer cells are gaining prominence as novel biomarkers for cancer monitoring and evaluating treatment efficacy. Their broad presence, ease of clinical detection, and the protection of encapsulated proteins from enzymatic degradation in the bloodstream make them valuable candidates for this purpose [11–13].

CA125 (Cancer Antigen 125, also known as MUC16) has long been utilized in the monitoring of ovarian cancer. Elevated CA125 levels may occur in certain benign conditions such as peritonitis, liver cirrhosis, endometriosis, and others [14, 15], making its specificity and sensitivity in the initial diagnosis and differentiation of benign and malignant ovarian masses suboptimal. Human Epididymis Protein 4 (HE4) has been identified as another promising biomarker that, in combination with CA125, can improve diagnostic accuracy [16, 17]. The complement system is a group of proteins present in body fluids and on the surface of cells. Once activated, it exhibits biological activity and participates in immune and inflammatory responses. It plays a significant role in the occurrence and development of tumors. This role is dual in nature, as the complement system has both an immune surveillance effect on tumors and the potential to promote tumor development, angiogenesis, invasion, and metastasis through excessive complement activation [18, 19]. Complement component 5a (C5a), a less explored biomarker, is a part of the complement system which has been implicated in the regulation of tumor progression. Ding demonstrated that intracellular complement C5a/C5aR1 stabilizes β-catenin [20], promoting colorectal tumorigenesis. This finding suggests that complement activation can contribute to the development of cancer by influencing key signaling pathways involved in tumor growth. Similarly, Hu and Maeda revealed that C5a receptor (C5aR) enhances invasiveness in hepatocellular carcinoma and metastatic renal cell carcinoma [21, 22], respectively, through the activation of ERK1/2-mediated pathways. These studies highlight the potential role of complement in promoting cancer cell invasion and metastasis. In the context of ovarian cancer, Zhang [23] demonstrated that C5aR1 blockade reshapes the immunosuppressive tumor microenvironment in high-grade serous ovarian cancer, suggesting that targeting complement signaling could enhance the effectiveness of immune checkpoint blockade therapy. Additionally, Nunez-Cruz and Cho provided evidence of the involvement of complement in ovarian cancer neovascularization and the autocrine effects of tumor-derived complement [24, 25], respectively, further emphasizing the significance of complement in ovarian cancer development and progression.

This study is dedicated to investigating the clinical importance of exosomal markers CA125, HE4, and C5a in the differentiation of benign and malignant ovarian tumors.

## Materials and methods

### Patients

This study gathered a cohort of 234 patients diagnosed with ovarian tumors through postoperative pathology examinations at the First Affiliated Hospital of Zhengzhou University from December 2019 to June 2021. Patient data including age, menstrual status, preoperative tumor markers, postoperative pathology type, and stage were meticulously recorded and documented. Among the 234 patients, 190 were placed in the benign group, while 44 patients were categorized into the non-benign group. Within the non-benign group, there were 10 cases of ovarian junctional tumors, and the remaining 34 cases were classified as malignant tumors. Among the 34 patients with pathologically confirmed malignant ovarian tumors, there were 29 cases of epithelial ovarian tumors including 18 high-grade plasmacytoid carcinomas, 1 case of moderately to poorly differentiated supportive-mesenchymal stromal cell tumor, 3 cases of adult-type granulosa cell tumor, and 1 case of squamous cell carcinoma. The staging of ovarian malignant tumors was determined according to the International Federation of Gynecology and Obstetrics (FIGO) criteria. Among the cases, there were 13 in stage I, 5 in stage II, 7 in stage III, and 9 in stage IV. Out of the 44 patients in the non-benign group, a total of 10 were diagnosed with junctional tumors. These included 4 cases of plasmacytoid tumors and 6 cases of mucinous tumors. Inclusion criteria for this study encompassed patients aged 18 years or older presenting with adnexal masses requiring surgical intervention. Exclusion criteria comprised individuals with a history of prior radiotherapy treatment, a previous malignancy, or those currently experiencing a pregnancy. The study obtained an exemption from requiring informed consent from the participants following a comprehensive review and approval by the Ethics Committee.

### Research methodology and judgement criteria

All study participants provided venous blood samples during the early morning fasting state. Serum CA125 and HE4 values were quantified using enzyme-linked immunosorbent assay (ELISA), and the ROMA index was computed using ROMA analysis software (CanAg Diagnostics AB).

Calculation formula: ROMA (%) = [exp(PI)*100]/[1 + exp( PI)] premenopausal predictive index (PI) = -12.0 + 2.38* LN (HE4) + 0.0626 *LN (CA125); postmenopausal predictive index (PI) = -8.09 + 1.04* LN (HE4) + 0.732* LN (CA125).

In the clinical context, the reference value for CA125 was established as less than 35 U/mL. For premenopausal patients, the reference value for HE4 was less than 68.96 pmol/L, while for postmenopausal patients, it was less than 114.9 pmol/L. (Expert consensus on the application of gynecological tumor markers, China, 2018). In the clinical context, the reference values for ROMA were as follows: less than 11.4% for premenopausal patients and 29.9% for postmenopausal patients. The levels of CA125, HE4, and C5a in the serum exosomes of the subjects were assessed using the Exosome Ovarian Cancer Auxiliary Diagnostic Kit. (chemiluminescence method) provided by Shanghai Sidi Biomedical Technology Co. The scores, denoted as OCS values, were subsequently analyzed using a calculation formula integrated into the instrument. Ultimately, the Receiver Operating Characteristic (ROC) curve was employed to establish the optimal cutoff value, where an OCS value of ≥ 0.497 was considered positive, while < 0.497 was deemed negative.

### Observation indicators

To assess the effectiveness of CA125, HE4, C5a, and OCS values within serum exosomes, as well as serum CA125, HE4, and ROMA indices, in distinguishing between benign and non-benign ovarian tumors, with pathological diagnosis as the gold standard, the formulas for each diagnostic index in the discrimination of these groups were as follows:

- Sensitivity = True Positive / (True Positive + False Negative).
- Specificity = True Negative / (True Negative + False Positive).
- Positive Predictive Value (PPV) = (True Positive / (True Positive + False Positive)) * 100%.
- Negative Predictive Value (NPV) = (True Negative / (True Negative + False Negative)) * 100%.
- Youden’s Index = Sensitivity + Specificity – 1.

Diagnostic efficacy was assessed by constructing Receiver Operating Characteristic (ROC) curves for each study subject, and the corresponding Area Under the Curve (AUC) was calculated. The ROC curve and AUC are common tools used to evaluate the accuracy of diagnostic tests or markers.

### Follow-up information

The initiation of the follow-up process was defined as the moment when the patient received a diagnosis of ovarian malignancy. The follow-up procedures involved contacting the patient by phone or through their visits to our outpatient clinic. The most recent follow-up took place on August 30, 2023. Tumor recurrence was deemed to occur when there was histopathological evidence obtained through biopsy and/or new findings on imaging subsequent to the completion of the patient’s treatment. Disease progression-free survival time was defined as the duration from the date of the initial disease diagnosis (confirmed through surgical or biopsy histopathology) to the occurrence of disease recurrence, patient mortality, or, in the event of loss to follow-up, the time of the last follow-up visit prior to that loss of contact.

### Statistical analyses

Data analysis was performed using SPSS 22.0 statistical software. The levels of CA125, HE4, and C5a in serum extracellular vesicles, as well as the levels of serum CA125, HE4, and ROMA indices, were represented as mean ± standard deviation (χ ± s). For normally distributed data, the t-test was employed, while the Mann-Whitney test was utilized for non-normally distributed data. Analysis of Variance (ANOVA) was conducted to compare the data across multiple groups. Additionally, Receiver Operating Characteristic (ROC) curves were generated for each index, and the Area Under the Curve (AUC) was calculated to assess their diagnostic performance. To compare the sensitivity and specificity in distinguishing between benign and non-benign groups, the McNemar test was employed, with statistical significance considered when *P* < 0.05. Survival analyses were conducted using the Kaplan-Meier method for one-way analyses and Log-rank tests. A Cox regression model was utilized for multifactorial prognosis analysis. The significance level was set at α = 0.05 (two-tailed).

## Results

### Comparison of marker levels within exosomes and serum in the non-benign and benign group

The mean age of the benign group was 34.78 ± 11.35 years, while that of the non-benign group was 42.91 ± 10.93years, and this difference was statistically significant (t = 4.441, *P* < 0.001).To determine whether there were differences in the levels of diagnostic indexes between the benign and non-benign groups, we employed an independent samples t-test. The results indicated that the mean values of serum CA125, HE4, ROMA index, extracellular vesicles CA125, HE4, C5a, and OCS exhibited statistically significant differences between the benign and non-benign groups at a significance level of 0.05. Upon further comparison, it was evident that the levels of all these indexes were higher in the non-benign group compared to the benign group. (Table 1)

**Table 1:** General information of patients

Item		Totaldata	Pathology		T	*P*
			Benign group	Non-benign group		
Age		36.162 ± 11.749	34.784 ± 11.348	42.910 ± 10.929	4.441	< 0.0001
Serum	CA125(U/mL)	80.322 ± 138.873	46.231 ± 68.991	260.343 ± 249.626	4.968	< 0.0001
	HE4(pmol/L)	79.633 ± 144.756	50.235 ± 12.287	248.770 ± 335.300	3.452	< 0.0001
Serum sEVs	OCS	0.306 ± 0.259	0.229 ± 0.179	0.640 ± 0.286	9.125	< 0.0001
	CA125(U/mL)	26.037 ± 62.415	9.226 ± 21.238	98.629 ± 111.739	5.285	< 0.0001
	HE4(pmol/L)	3.473 ± 9.190	1.900 ± 0.751	10.269 ± 19.925	2.786	< 0.0001
	C5a(ng/ml)	4.902 ± 2.572	4.454 ± 2.188	6.837 ± 3.178	4.720	< 0.0001
ROMA idex		14.600 ± 19.523	9.064 ± 6.043	44.520 ± 36.839	5.594	< 0.0001

### Exosomes and serum protein levels between different benign ovarian tumours

To explore variations in serum CA125, HE4, ROMA index, and Serum sEV levels within the subcategories of benign diseases, we further divided the benign group into four subgroups: ovarian endometriosis group (73 cases), mature teratoma group (48 cases), cystadenoma group (34 cases), and other benign tumors group (including luteal cysts, luteinized cysts, follicular cysts, haemorrhagic cysts, etc., totaling 35 cases). The outcomes of one-way analysis of variance (ANOVA) indicated that age [F (3, 186) = 10.144, *p* < 0.001] and serum CA125 levels (F(3, 186) =, *p* < 0.001) exhibited significant differences among the benign disease categories. However, the levels of other indicators did not show significant differences within the benign groups at the 0.05 significance level. Notably, serum CA125 levels were notably higher in the ovarian endometriosis group compared to the mature teratoma group and the other tumor groups, respectively. Additionally, the mean age of the other benign tumor group was greater than that of the ovarian endometriosis and mature teratoma groups (Table 2).

**Table 2:** General information on patients in each subgroup of the benign and non-benign groups

Item		Benign group						Non-benign group				F	*P*
		Endometriosis cysts	Cystadenoma	Mature teratoma	Other neoplasms	F1	*P*1	BOTs	OC	Z	*P2*		
Age		33.92 ± 7.94	35.12 ± 13.78	30.00 ± 9.87	42.83 ± 12.76	10.144	< 0.001	32.90 ± 11.56	45.85 ± 8.938	14.183	0.011	13.036	< 0.001
Exosome	OCS	0.271 ± 0.196	0.199 ± 0.181	0.188 ± 0.118	0.226 ± 0.199	2.594	0.054	0.521 ± 0.220	0.674 ± 0.296	1.51	0.12	32.057	< 0.001
	CA125(U/mL)	10.99 ± 13.31	13.82 ± 41.66	3.43 ± 2.89	9.03 ± 18.99	1.920	0.128	32.65 ± 57.97	118.04 ± 116.81	2.219	0.026	27.888	< 0.001
	HE4(pmol/L)	1.87 ± 0.81	1.86 ± 0.63	1.94 ± 0.66	1.94 ± 0.86	0.129	0.943	3.71 ± 1.84	12.20 ± 22.35	2.191	0.624	8.395	< 0.001
	C5a(ng/ml)	4.73 ± 2.66	3.98 ± 1.48	4.62 ± 2.04	4.13 ± 1.79	1.267	0.287	5.85 ± 3.24	7.13 ± 3.15	1.119	0.11	8.136	< 0.001
ROMAindex		9.26 ± 5.64	10.82 ± 8.34	7.51 ± 4.29	9.26 ± 6.01	2.093	0.103	17.43 ± 9.71	44.52 ± 36.84	3.857	0.13	31.816	< 0.001
Serum	CA125(U/mL)	75.25 ± 83.00	36.79 ± 77.08	18.67 ± 12.71	32.75 ± 52.25	8.386	< 0.001	115.99 ± 149.92	260.34 ± 249.63	1.730	0.134	21.560	< 0.001
	HE4(pmol/L)	51.35 ± 9.75	52.94 ± 17.90	47.11 ± 10.22	49.57 ± 12.58	1.840	0.141	63.13 ± 21.49	248.77 ± 335.30	1.735	0.005	13.897	< 0.001

BOTs indicates Borderline Ovarian Tumors group; OC indicates Ovarian Cancer group;

### Comparison of serum exosomes and serum protein levels in the non-benign group

Within the non-benign ovarian tumor group, we further subdivided it into the borderline tumor group and the malignant tumor group. Initially, we conducted the Shapiro-Wilk test on the data from both groups. At a significance level of α = 0.05, the data for each index did not adhere to a normal distribution. Consequently, we applied the Mann-Whitney test to both sets of data. The test outcomes revealed that age, serum sEV CA125 level, and serum HE4 level were all significantly higher in the ovarian malignant tumor group compared to the ovarian borderline tumor group (Table 2).

### Comparison of serum exosomes and serum protein levels in all groups

We employed ANOVA to make comparisons among the four benign groups and the borderline, malignant, and non-benign groups, respectively. The results of post-hoc multiple tests indicated that, with the exception of the level of HE-4 in extracellular vesicles, the ovarian malignant and non-benign groups exhibited higher mean values for serum CA125, HE4, ROMA index, as well as mean values for CA125, C5a, and OCS in serum sEV, compared to the levels of these indicators observed within the four benign groups.

### Correlation and differences in the determination of CA125 and HE4 levels between serum exosomes and serum

As presented in Table 3, we conducted an analysis of the levels of CA125 and HE4 in paired serum exosomes and serum samples from the same patients using the paired t-test. The results of Pearson correlation analysis indicated that, across all data, the benign group, and the non-benign group, there was a positive correlation between serum sEV and serum levels of CA125 (with correlation coefficients of 0.805, 0.697, and 0.749, respectively, all with *P* < 0.05) as well as HE4 levels (with correlation coefficients of 0.752, 0.281, and 0.713, respectively, all with *P* < 0.05). It is noteworthy that both in the overall dataset and in the benign and non-benign groups, the levels of serum sEV CA125 and HE4 were lower than those in the serum samples, and this difference was statistically significant at the 0.05 significance level.

**Table 3:** Comparison of CA125 and HE4 levels in serum sEV and serum pairs

		r	Mean	Standard Deviation	t	*P*
Total data	S-CA125&E-CA125	0.805	54.286	96.079	8.643	< 0.001
	S-HE4&E-HE4	0.752	76.160	137.981	8.443	< 0.001
Benign group	S-CA125&E-CA125	0.697	37.01	56.29	9.061	< 0.001
	S-HE4&E-HE4	0.281	48.34	12.10	55.076	< 0.001
Non-benign group	S-CA125&E-CA125	0.749	128.91	170.51	5.015	< 0.001
	S-HE4&E-HE4	0.713	196.31	290.39	4.484	< 0.001

r enotes correlation coefficient

### Differential diagnosis of benign and non-benign ovarian tumours by serum exosomes and serum proteins

The sensitivity and specificity of serum CA125, serum sEV CA125, HE4, C5a, and their combination (OCS) for differentiating between benign and non-benign ovarian tumors were as follows: serum CA125 sensitivity was 75%, specificity was 66.8%; serum sEV CA125 sensitivity was 61.4%, specificity was 96.3%; HE4 sensitivity was 77.3%, specificity was 73.7%; C5a sensitivity was 68.2%, specificity was 77.9%; OCS sensitivity was 77.3%, and specificity was 93.2%.

Serum HE4 levels and the ROMA index are influenced by women’s menstrual status. Therefore, we divided the non-benign group into premenopausal and postmenopausal subgroups to evaluate the diagnostic ability of these two indices for distinguishing between benign and malignant ovarian tumors in both groups. In Table 4, it can be observed that in premenopausal women, the sensitivity of the ROMA index (≥ 11.4) and serum HE4 (> 68.96 pmol/L) were 60.7% and 57.1%, respectively, with specificities of 95.3% and 80.6%. In postmenopausal women, the sensitivity of the ROMA index (≥ 29.9) and serum HE4 (> 114.9 pmol/L) was 73.3% and 66.7%, respectively, with specificities of 100% and 85%. Notably, the sensitivity and specificity of serum HE4 and the ROMA index in the differential diagnosis of benign and non-benign ovarian tumors in postmenopausal women were higher than those in premenopausal women.

**Table 4:** Diagnostic efficacy of various indicators on ovarian cancer

Item		SE(%)	SP(%)	PPV(%)	NPV(%)	Accuracy(%)	Youden index(%)
Serum	CA125(U/mL)	75	66.8	34.4	92.0	70.9	41.8
	HE4(pmol/L)	63.6	95.8	77.8	91.9	79.7	59.4
	Premenopausal	57.1	95.3	66.7	93.1	76.2	52.4
	Postmenopausal	73.3	100	100	83.3	86.65	73.3
Serum sEVs	OCS	77.3	93.2	72.3	90.9	85.25	70.5
	CA125(U/mL)	61.4	96.3	75	91.4	78.85	57.7
	HE4(pmol/L)	77.3	73.7	40,5	93.3	75.5	51
	C5a(ng/ml)	68.2	77.9	41.7	91.4	73.05	46.1
ROMA		63.6	81.1	43.8	90.6	72.35	44.7
Premenopausal		60.7	80.6	34.0	92.6	70.65	41.3
Postmenopausal		66.7	85	76.9	77.3	75.85	51.7

### Comparison of AUC of serum sEV and serum proteins

**Figure 1:**
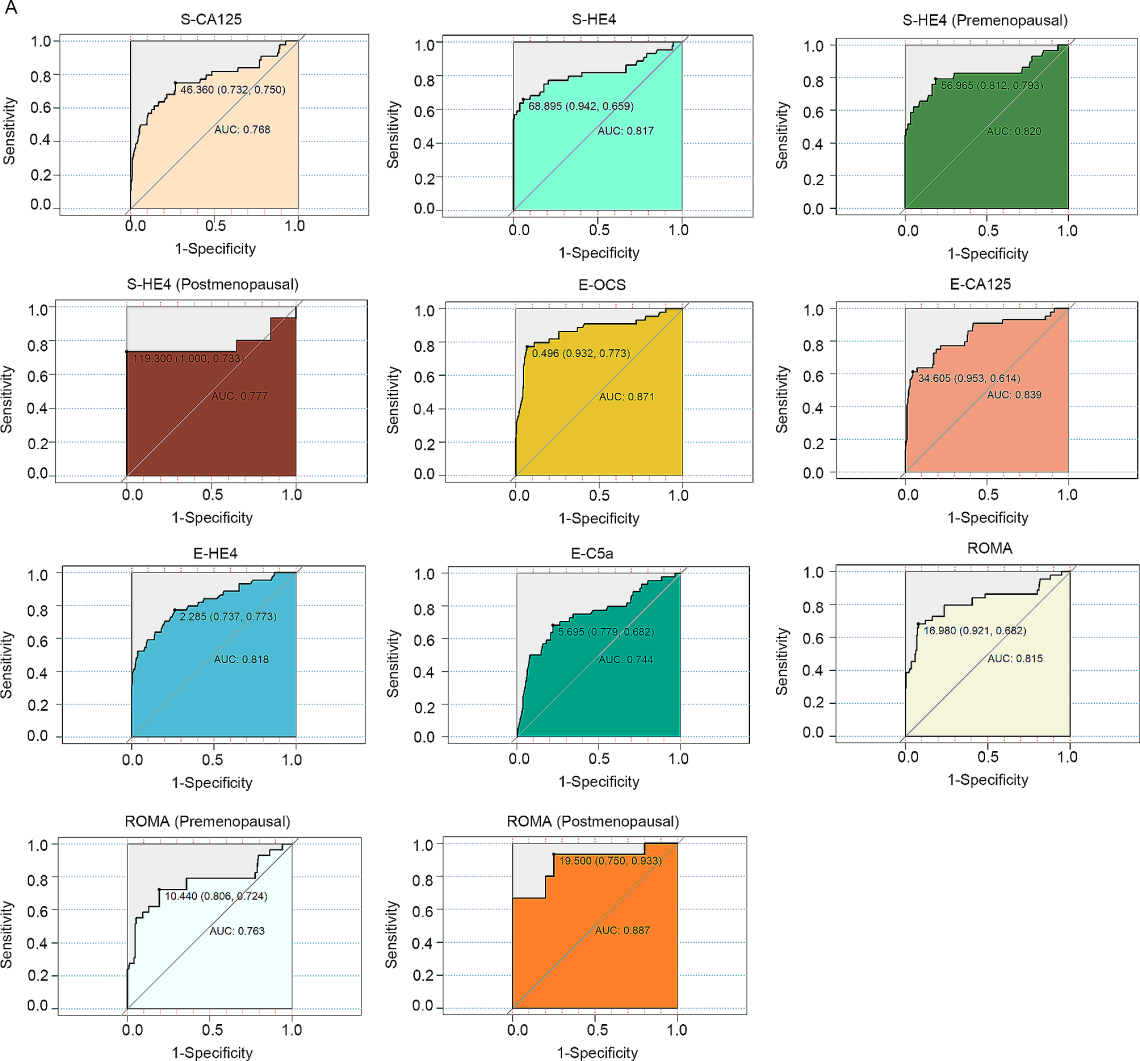
ROC curve of ovarian malignancy diagnosed by various indicators. **A**, ROC curve of ovarian malignancy diagnosed by various indicators

We evaluated the ability to distinguish between benign and non-benign ovarian tumors using the AUC of CA125, HE4, and C5a within serum sEV, as well as the combined index OCS with serum CA125, HE4, and ROMA indices. The AUC values for serum CA125, HE4, and ROMA index were 0.786, 0.817, and 0.815, respectively. For serum CA125, HE4, and C5a within serum sEV, as well as the combined index (OCS), the AUC values were 0.839, 0.818, 0.744, and 0.871, respectively (Fig. 1). To further assess the diagnostic efficacy of the ROMA index and serum HE4 for distinguishing between benign and non-benign ovarian tumors in pre and postmenopausal women, we subdivided the non-benign group into these two groups. In Table 5, we observed that the AUCs for the ROMA index (≥ 11.4) and serum HE4 (> 68.96 pmol/L) were 0.815 and 0.777 in premenopausal women, and 0.777 and 0.887 in postmenopausal women for ROMA index (≥ 29.9) and serum HE4 (> 114.9 pmol/L).To determine whether there were significant differences in sensitivity and specificity between different methods and indexes, we conducted McNemar tests to compare the differential diagnostic indexes two by two. The results indicated that the discriminative ability of the OCS index and extracellular vesicle CA125 was superior to that of serum CA125 (*P* < 0.05). Additionally, the discriminative ability of OCS was better than that of extracellular vesicle HE4 and C5a (*P* < 0.05), while the other two-by-two comparisons did not demonstrate any statistically significant differences (Table 6).

**Table 5:** ROC curve analysis of ovarian malignancy in the diagnosis of various indicators

Item		AUC	Standard error	Cutoff	*P*	95%CI	
						Lower limit	Upper limit
Serum	CA125(U/mL)	0.768	0.047	46.360	< 0.0001	0.675	0.860
	HE4(pmol/L)	0.817	0.04	68.895	< 0.0001	0.725	0.908
	Premenopausal	0.820	0.0567	56.965	< 0.0001	0.760	0.871
	Postmenopausal	0.777	0.102	119.300	< 0.0001	0.604	0.899
Serum sEVs	OCS	0.871	0.037	0.497	< 0.0001	0.799	0.943
	CA125(U/mL)	0.839	0.039	34.605	< 0.0001	0.763	0.915
	HE4(pmol/L)	0.818	0.040	2.285	< 0.0001	0.740	0.896
	C5a(ng/ml)	0.744	0.046	5.695	< 0.0001	0.654	0.835
ROMA		0.815	0.045	16.980	< 0.0001	0.728	0.902
Premenopausal		0.763	0.061	10.440	< 0.0001	0.698	0.821
Postmenopausal		0.887	0.0619	19.500	< 0.0001	0.734	0.968

**Table 6:** Comparison of the AUC of each index for the diagnosis of ovarian tumours

		Serum		Serum sEVs				ROMA
		CA125(U/mL)	HE4(pmol/L)	OCS	CA125(U/mL)	HE4(pmol/L)	C5a(ng/ml)	
Serum	CA125(U/mL)	-	0.1506	0.0067	0.0034	0.1421	0.6932	0.2154
	HE4(pmol/L)	0.1506	-	0.1421	0.5097	0.9699	0.2056	0.9523
Exosome	OCS	0.0067	0.1421	-	0.1569	0.0409	0.0009	0.1668
	CA125(U/mL)	0.0034	0.5097	0.1569	-	0.6077	0.0371	0.5136
	HE4(pmol/L)	0.1421	0.9699	0.0409	0.6077	-	0.1149	0.9396
	C5a(ng/ml)	0.6932	0.2056	0.0009	0.0371	0.1149	-	0.2306
ROMA		0.2154	0.9523	0.1668	0.5136	0.9396	0.2306	-

### Analysis of prognostic factors in malignant tumours of the ovary

The median follow-up period was 27.9 months, ranging from 8.1 to 41.9 months. Among the 34 cases, 7 patients passed away, 3 experienced disease relapse, 24 survived without disease progression, and 3 were lost to follow-up. The 1-year progression-free survival rate was 94.1%, and the 3-year disease-free survival rate was 73.5%.We conducted a univariate analysis to examine the impact of various factors, including age, menstrual status, pathological type, stage, extracellular vesicular intravesicular CA125, HE4, C5a, and the combined index OCS with serum CA125, HE4, and ROMA index, on the prognosis of ovarian malignancy using the Kaplan-Meier method. The results indicated that tumor stage and serum HE4 level may be associated with the prognosis of ovarian malignancy at a significance level of 0.05 (Table 7). Subsequently, we performed Cox regression analysis specifically on these two individual factors. The results, as displayed in Table 7, confirmed that tumor stage was an independent risk factor influencing the survival of patients with ovarian malignancy (*p* = 0.043) (Table 8).

**Table 7:** Prognostic unifactorial analysis of 34 patients with ovarian malignancy

	Considerations	N(%)	Χ2	P		Considerations	N(%)	Χ2	P
Age(y)	>50	14(64.3%)	0.657	0.417	E-C5a(ng/ml)	>7.4	17(64.7%)	1.285	0.257
	≤ 50	20(75.0%)				≤ 7.4	17(76.5%)		
menopausal	Premenopausal	13(69.2%)	0.076	0.782	OCS	>0.600	22(83.3%)	3.509	0.061
	Postmenopausal	21(71.4%)				≤ 0.600	12(63.6%)		
grade	I ~ II	18(88.9%)	5.012	0.025	S-CA125(U/ml)	>300	14(64.3%)	0.342	0.559
	III ~ IV	16(50.0%)				≤ 300	20(75.0%)		
physiology	Epithelial	29(69.0%)	0.396	0.529	S-HE4(pmol/L)	a	20(60.0%)	4.007	0.045
	others	5(80.0%)				b	14(85.7%)		
E-CA125(U/ml)	>125	13(84.6%)	1.613	0.204	ROMA(%)	c	18(66.7%)	0.624	0.430
	≤ 125	21(61.9%)				d	16(75.0%)		
E-HE4(pmol/L)	>3.5	15(73.3%)	0.106	0.744					
	≤ 3.5	19(68.4%)							

- a: Serum HE4 levels greater than 100 pmol/L in premenopausal women or greater than 170 pmol/L in postmenopausal women

- b: Serum HE4 levels less than or equal to 100 pmol/L in premenopausal women or less than or equal to 170 pmol/L in postmenopausal women

- c: ROMA index greater than 20 in premenopausal women or greater than 40 in postmenopausal women

- d: ROMA index less than or equal to 20 in premenopausal women or less than or equal to 40 in postmenopausal women

**Table 8:** Multifactorial analysis of the prognosis of 34 patients with ovarian malignancy

	B	SE	Wald	p	Exp(B)	95.0%CI
grade	1.603	0.793	4.084	0.043	4.968	1.049 ~ 23.516
S-HE4	1.852	1.062	3.042	0.081	6.372	0.795 ~ 51.067

## Discussion

In this study, we conducted a quantitative analysis of CA125, HE4, C5a, and their combined indicators within serum exosomes. We evaluated the utility of these serum exosomes proteins for distinguishing between benign and non-benign ovarian tumors by comparing them with traditional serum tumor markers. This approach allowed us to assess the potential diagnostic value of these markers in a more comprehensive and detailed manner, aiming to improve the accuracy of ovarian tumor diagnosis.

This paper’s findings regarding elevated serum exosomes protein levels in patients with non-benign tumors align with previous proteomics research. These results are consistent with prior studies that have suggested a correlation between specific protein markers and the presence of malignant ovarian tumors [26, 27]. Moreover, previous research has demonstrated elevated concentrations of total exosomes in serum samples from ovarian cancer patients. Exosomes have also been detected in various body fluids, including saliva, plasma, urine, ascites, and cerebrospinal fluid. These findings provide a solid foundation for considering extracellular intravesicular proteins as potential new tumor markers [27, 28]. These exosomal proteins have the potential to become novel biomarkers that open up new possibilities and prospects for early detection and monitoring of ovarian malignancies. Early detection is crucial in the diagnosis and treatment of cancer, and the cure and survival rates of patients are usually significantly improved by intervening in the early stages of the disease. Therefore, if these exosomal proteins can be accurately detected in the early stages of ovarian malignancy, they could serve as a powerful tool to help us identify the disease earlier and thus begin treatment sooner. In addition, these exosomal proteins may also be used to monitor disease progression and treatment response in ovarian malignancies. This means that by measuring the levels of these proteins on a regular basis, we may be able to track the progression of the disease, assess the patient’s response to treatment, and predict the prognosis of the disease.

Chen’s study has concluded that CA125 is detected at higher levels in exosomes compared to serum [26]. Contrary to Chen’s findings, the results from LI’s study suggested that the concentration of CA125 in exosomes was lower than that in serum [29]. We similarly compared the differences in intra-exosomal and serum mid-pair CA125 and HE4 levels in different subgroups of ovarian tumour patients. The results showed that there was a positive correlation between intra-exosomal and serum CA125 and HE4 levels, and the serum intra-exosomal CA125 and HE4 levels were higher than the intra-exosomal CA125 and HE4 levels. There is a certain correlation between the levels of CA125 and HE4 in exosomes and those in serum, which may imply that the levels of CA125 and HE4 in exosomes can serve as an indicator reflecting the presence of these biomarkers in serum. In addition, the observation that the levels of CA125 and HE4 in serum are higher than those in exosomes suggests that there may be other factors or components in the serum that may interact with CA125 and HE4, resulting in higher levels in the serum than in exosomes. We need to further investigate the specific mechanisms of the observed differences between the levels in exosomes and serum.

Menopause can impact the effectiveness of HE4 levels and the ROMA index in distinguishing between ovarian tumors [30, 31]. In this study, it was observed that the sensitivity and specificity of serum HE4 and the ROMA index were more favorable in postmenopausal patients compared to premenopausal ones. However, it’s important to note that the limited number of postmenopausal cases included may introduce bias to these findings. Based on the ROC curve analysis and taking pathological diagnosis as the reference standard, it appears that the exosomal OCS values generally exhibit better sensitivity, specificity, positive predictive value, and negative predictive value compared to other diagnostic indexes. In this study, the combination of exosomal markers showed superior efficacy in distinguishing between benign and non-benign tumors compared to serum tumor markers. Indeed, the findings suggest that exosomal OCS values have promising potential for the differential diagnosis of ovarian tumors. Additionally, the higher AUC of CA125 and HE4 in extracellular vesicles compared to the serum group underscores the value of examining these markers within exosomes for early-stage detection of ovarian cancer. This aligns with previous research by Li et al., which also highlighted the superiority of serum exosomal markers in identifying early-stage EOC patients compared to serum markers alone. These results emphasize the potential clinical utility of examining markers within exosomes for improved ovarian cancer diagnosis and management [29].

Our conclusion is consistent with the findings discussed earlier, highlighting the potential benefits of combining serum exosomal CA125, HE4, and C5a for the differential diagnosis of benign and non-benign ovarian tumors. This combination appears to offer improved sensitivity and specificity compared to single serum markers like HE4, CA125, and the ROMA index. Such an approach holds promise for enhancing early screening and diagnosis of ovarian tumors, which can ultimately contribute to improved patient outcomes and more effective clinical management.

We’ve correctly identified some limitations in the studies discussed. Indeed, small sample sizes and the single-center nature of the study can limit the generalizability of findings. Uneven distribution of patients in the cancer and benign tumour groups may cause statistical bias. Larger, multi-center studies with more diverse populations can provide more robust and representative results. Additionally, the field of medical research is continually evolving, and further investigations and studies are essential to validate and build upon the initial findings. Researchers often address these limitations by conducting follow-up studies or collaborating with multiple institutions to expand sample sizes and enhance the reliability of their results.

## Data Availability

The raw data supporting the conclusions of this article will be made available by the authors without undue reservation.
